# Post-exercise Hypotension Following a Single Bout of High Intensity Interval Exercise vs. a Single Bout of Moderate Intensity Continuous Exercise in Adults With or Without Hypertension: A Systematic Review and Meta-Analysis of Randomized Clinical Trials

**DOI:** 10.3389/fphys.2021.675289

**Published:** 2021-06-28

**Authors:** Isabela Roque Marçal, Karla Fabiana Goessler, Roselien Buys, Juliano Casonatto, Emmanuel Gomes Ciolac, Véronique A. Cornelissen

**Affiliations:** ^1^Exercise and Chronic Disease Research Laboratory, Department of Physical Education, School of Sciences, São Paulo State University (UNESP), Bauru, Brazil; ^2^Research Group for Cardiovascular Rehabilitation, Department of Rehabilitation Sciences, University of Leuven, KU Leuven, Leuven, Belgium; ^3^Applied Physiology and Nutrition Research Group, School of Physical Education and Sport, Faculty of Medicine (FMUSP), University of Saõ Paulo, Saõ Paulo, Brazil; ^4^Research Group in Physiology and Physical Activity, University of Northern Paraná, Londrina, Brazil

**Keywords:** post-exercice, hypotension, high intensity interval exercise, systematic review & meta-analysis, office blood pressure, ambulatory blood pressure, moderate intensity aerobic exercise

## Abstract

**Background:** Post-exercise hypotension (PEH) is an important tool in the daily management of patients with hypertension. Varying the exercise parameters is likely to change the blood pressure (BP) response following a bout of exercise. In recent years, high-intensity interval exercise (HIIE) has gained significant popularity in exercise-based prevention and rehabilitation of clinical populations. Yet, to date, it is not known whether a single session of HIIE maximizes PEH more than a bout of moderate-intensity continuous exercise (MICE).

**Objective:** To compare the effect of HIIE vs. MICE on PEH by means of a systematic review and meta-analysis.

**Methods:** A systematic search in the electronic databases MEDLINE, Embase, and SPORTDiscus was conducted from the earliest date available until February 24, 2020. Randomized clinical trials comparing the transient effect of a single bout of HIIE to MICE on office and/or ambulatory BP in humans (≥18 years) were included. Data were pooled using random effects models with summary data reported as weighted means and 95% confidence interval (CIs).

**Results:** Data from 14 trials were included, involving 18 comparisons between HIIE and MICE and 276 (193 males) participants. The immediate effects, measured as office BP at 30- and 60-min post-exercise, was similar for a bout of HIIE and MICE (*p* > 0.05 for systolic and diastolic BP). However, HIIE elicited a more pronounced BP reduction than MICE [(−5.3 mmHg (−7.3 to −3.3)/ −1.63 mmHg (−3.00 to −0.26)] during the subsequent hours of ambulatory daytime monitoring. No differences were observed for ambulatory nighttime BP (*p* > 0.05).

**Conclusion:** HIIE promoted a larger PEH than MICE on ambulatory daytime BP. However, the number of studies was low, patients were mostly young to middle-aged individuals, and only a few studies included patients with hypertension. Therefore, there is a need for studies that involve older individuals with hypertension and use ambulatory BP monitoring to confirm HIIE's superiority as a safe BP lowering intervention in today's clinical practice.

**Systematic Review Registration:** PROSPERO (registration number: CRD42020171640).

## Introduction

Hypertension remains the most important modifiable risk factor for cardiovascular morbidity and mortality (Forouzanfar et al., 2016). In international studies, the rate of elevated systolic blood pressure (BP) (≥110–115 and ≥140 mm Hg) increased substantially between 1990 and 2015, and disability-adjusted life years and deaths associated with elevated BP also increased (Forouzanfar et al., 2017). With the aging of the population, a further increase of 15–20% is expected worldwide (Williams et al., 2018).

To reduce the burden associated with hypertension, more emphasis on lifestyle changes is needed. Nowadays, all guidelines on BP management unequivocally recommend exercise as an important non-pharmacological therapy in the prevention, treatment, and control of high BP (Whelton et al., 2018; Williams et al., 2018; Hanssen et al., 2021). Preferably, exercise is performed on a daily basis, as it was previously shown that BP is significantly reduced following a single bout of exercise (Pescatello et al., 2004). If sustained and lasting long enough, this phenomenon-which is called post-exercise hypotension (PEH)-can play an important role in the daily management of hypertensive patients by transiently lowering their BP toward (more) normal values for a significant part of the day (MacDonald, 2002).

In line with pharmacokinetics of drug therapy, it might be expected that the occurrence and magnitude of PEH following a single bout or dose of exercise will depend on the exercise characteristics: i.e., type of exercise, volume, duration, or intensity of the session. Though, results on, for instance, the role of aerobic exercise intensity remains inconclusive. Pescatello and colleagues found PEH to be more pronounced in the first 5 h after a 40-min bout at moderate (60% of VO2max) vs. light intensity (40% of VO2max), though this difference disappeared when BP was measured over the full course of 9 h (Pescatello et al., 2004). In contrast, others found PEH to be larger after higher vs. lower intensity exercise bouts when PEH was evaluated by means of 24 h ambulatory BP monitoring (Quinn, 2000; Eicher et al., 2010). In recent years, growing evidence has shown that high-intensity interval training provokes superior health benefits compared to moderate-intensity continuous training in both healthy individuals and patients with established cardiovascular disease (Pattyn et al., 2014; Ito, 2019; Liu et al., 2019; Williams et al., 2019).

Contrary to these overall superior results, studies investigating the effect of high intensity interval training on BP have been less conclusive. A recent meta-analysis pooling data from seven trials (164 participants) found comparable reductions in office BP in adults with pre- to established hypertension following chronic high intensity interval training and moderate intensity continuous training (Costa et al., 2018). In line with this, similar changes in 24 h ambulatory BP (three studies, 93 participants) and measures of central arterial stiffness (13 studies, 395 participants) following 4–16 weeks of high intensity interval training or moderate intensity continuous training was found in another meta-analysis (Way et al., 2019). On the other hand, larger reductions in office diastolic BP, but not systolic BP, were reported after high intensity interval training in a meta-analysis of 15 studies including only patients with hypertension (Leal et al., 2020). However, whether a single bout of high intensity interval exercise (HIIE) affects PEH more than a bout of moderate intensity continuous exercise (MICE) remains to be determined as individual studies have been small and reported contradictory results (Tordi et al., 2010; Pimenta et al., 2019).

Therefore, the aim of this systematic review and meta-analysis was to assess the effect of a bout of HIIE vs. a bout of moderate intensity continuous exercise (MICE) on PEH in individuals with normal BP, pre-hypertension or hypertension.

## Methods

This systematic review was conducted and reported according to the Preferred Reporting Items of Systematic Reviews and Meta-Analyses (PRISMA) guidelines (Liberati et al., 2009). The study protocol was prospectively registered with PROSPERO (registration number: CRD42020171640).

### Search Strategy

A systematic search was performed in three electronic databases (MEDLINE [OvidSP], Embase [OvidSP], and SPORTDiscus [EBSCOhost]) from their inception to February 24, 2020. The search strategy included a combination of free text terms for the key concepts “blood pressure,” “high intensity interval exercise,” and “moderate intensity continuous exercise.” The full search strategy for each database search is shown in Supplementary File 1 in Supplementary Material. No language restrictions were imposed on the search.

### Study Eligibility Criteria

Studies were included if they applied a randomized clinical trial design and were performed in humans (≥18 years) with an optimal BP, normal BP, high normal BP, or hypertension, and with no other concomitant disease. Trials should compare the effect of one single session of land-based HIIE vs. one single session of land-based MICE and report on office and/or ambulatory BP measured at least 30 min following the exercise bouts. Only data from full-text peer-reviewed publications were considered for inclusion. Exclusion criteria included any study not meeting all the criteria above.

### Study Selection

Citations were imported into Rayyan, a specific electronic application for systematic review and meta-analysis (https://rayyan.qcri.org/welcome), and duplicates were identified and subsequently removed using the duplicate function. Then, two reviewers (I.R.M., K.F.G.) independently screened the titles and abstracts of all studies for eligibility. Then the full texts of all studies that met the inclusion criteria, or if there was uncertainty, were retrieved and reviewed by both reviewers. Disagreements between both reviewers were discussed with a third researcher (V.A.C) to obtain consensus. Reviewers were not blinded to the journal or authors. The rationale for excluding full-text articles was documented.

### Data Extraction

A specific developed data extraction file was used by both authors (I.R.M., K.F.G.) to extract data on study source (authors, publication year, country of origin), study design, sample size, participant characteristics (mean age, sex distribution, hypertension status), exercise intervention characteristics (intensity, duration, mode), BP assessment method, BP outcomes, and outcomes related to BP regulating mechanisms. Discrepancies were resolved by consensus. Authors of 13 studies were contacted twice by e-mail over a 1-month period asking to provide missing data in cases of incomplete reporting. After 1 month, five authors provided more detailed information (Mourot et al., 2004; de Carvalho et al., 2014; Morales-palomo et al., 2017; Pimenta et al., 2019), two authors reported no access to the data (Scott et al., 2008; Lacombe et al., 2011), one author reported the lack of these data (Klein et al., 2019), and five authors did not reply.

### Assessment of Study Quality

Two reviewers (I.R.M., K.F.G.) independently assessed the methodological quality by using the Cochrane Collaboration “risk of bias” tool (Review Manager 5.3). The Cochrane Risk of Bias Tool was slightly adapted to the study design and consisted of the following items: (i) random sequence generation, (ii) blinding of outcome assessment, (iii) incomplete outcome data for BP, (iv) eligibility criteria clearly described, (v) exercise intervention reproducible, (vi) point and variability measure reported for all BP measurements, and (vii) BP measured by automated device. Each criterion was rated by I.R.M. and K.F.G. as either “high risk,” “low risk,” or “unclear” risk of bias. In case of disagreement of rating, agreement was solved by mutual consensus. Studies were not excluded based on their quality.

### Statistical Analyses

Statistical analyses were performed with Comprehensive Meta-Analysis software (CMA, version 2.2.064, Biostat, NJ, USA). The primary outcome measures were responses in office (30 and 60 min post-exercise) and ambulatory systolic and diastolic BP. Data expressed using the standard error of the mean (SEM) were first converted to standard deviation (SD) by the formula: SD = SEM x √n. To compare the effects of single bouts of HIIE vs. MICE on office BP, we first calculated delta-score between post-exercise BP (at 30 or 60 min) and pre-exercise BP. Imputed study-level correlation coefficient for change from pre-intervention SD was set at a conservative estimate of 0.5 across all studies. For studies that compared two different HIIE study groups to a single MICE group, separate effect sizes were calculated for each comparison. To compare the effect of both exercise interventions on ambulatory BP we used the post intervention mean BP's following HIIE and MICE. Individual study results were then pooled using random-effect models with significance set at *p* < 0.05 (two-tailed). In addition, we also computed standardized mean difference (SMD), i.e., mean difference between the interventions divided by the pooled standard deviation. Descriptive data for each of the individual studies are reported as means ± standard deviations (SD); pooled effects are reported as mean weighted difference and its 95% confidence intervals (CI). *I*^2^ statistics were calculated to provide an estimation of the degree of heterogeneity in effect among the studies. *I*^2^ between 25 and 50% represents small amounts of inconsistency, whereas between 50 and 75% and above 75% represents medium to large amounts of inconsistency, respectively (Higgins and Thompson, 2002). Publication bias was examined by visual inspection of the different funnel plots' asymmetry. Duval and Tweedie's Trim and Fill procedure was applied to estimate the effect of publication bias on the results (Duval and Tweedie, 2000). In addition, small-study effect was investigated by regression of effect sizes and standard error of effect sizes as proposed by Egger et al. (1997). Finally, sensitivity analysis excluding selected trials which differed on a specific characteristic from the overall trials included in the analyses were performed to explore the robustness of results.

## Results

### Study Selection

**Figure 1:**
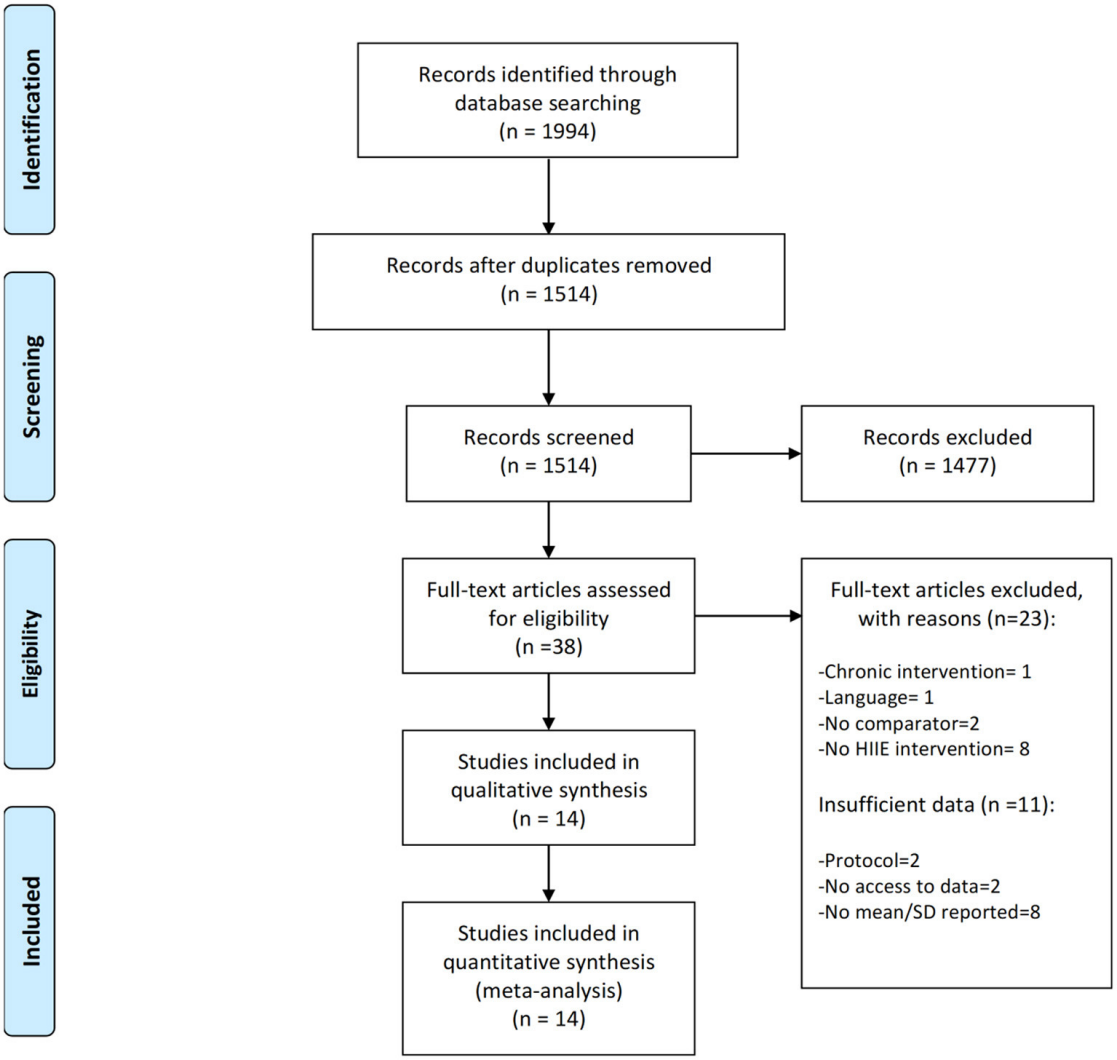
Prisma flow diagram depicting the study selection process.

A PRISMA flow diagram of the literature search and selection is presented in Figure 1. The initial search identified 1994 potentially relevant studies of which 37 full text articles were assessed for eligibility. After screening of the full-text, 14 papers could be included in the final meta-analysis. Three studies (Angadi et al., 2015; Morales-palomo et al., 2017; Ramirez-Jimenez et al., 2017) included multiple HIIE interventions or more than one patient group (i.e., a normotensive and hypertensive group). As a result, 18 comparisons were included in the final analysis.

### Risk of Bias Within and Across Studies

**Figure 2:**
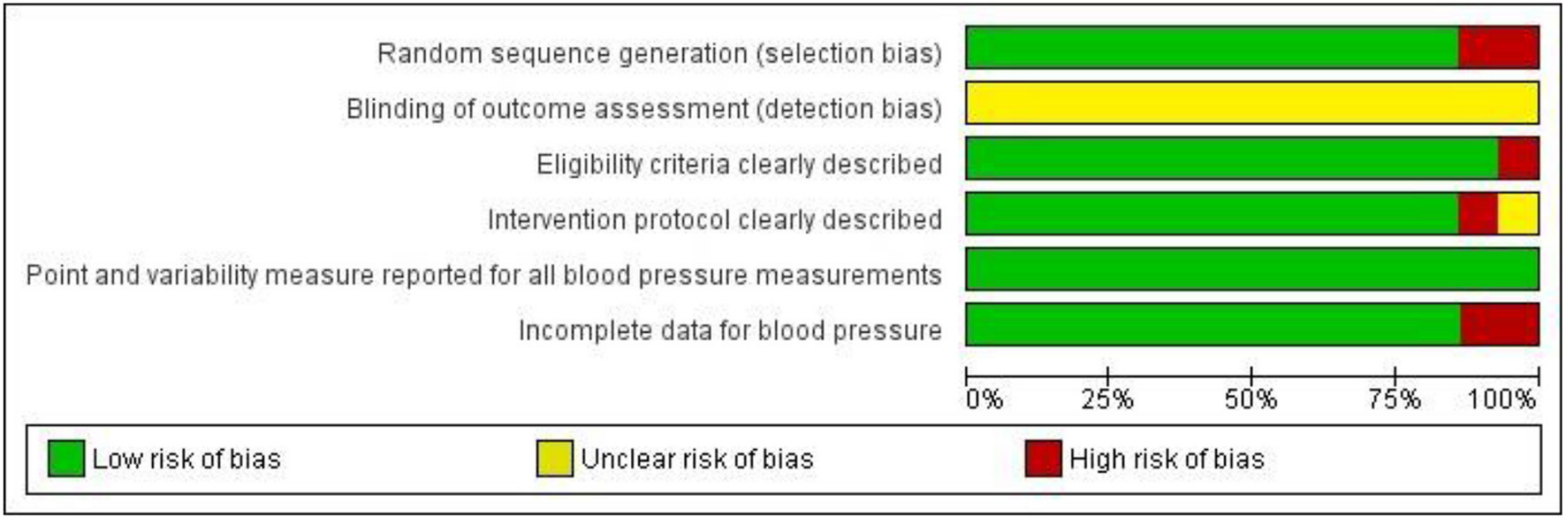
Summary of risk of bias for each item presented as a percentage across all included studies.

The risk of bias is depicted in Figure 2. The kappa correlation showed a good overall agreement between both reviewers (*k* = 0.656; 95% CI 0.680–0.852; *p* < 0.001). Two studies did not report on the randomization sequence (Mourot et al., 2004; Tordi et al., 2010) and one study lacked a proper description of the eligibility criteria (Angadi et al., 2015). None of the studies explicitly stated that researchers were blinded, and all studies were thus classified as unclear for the risk “blinding of outcome assessment.” Seven studies reported that office BP measurements were performed by an automated device (Rossow et al., 2009; Tordi et al., 2010; Angadi et al., 2015; Costa et al., 2016; Graham et al., 2016; Morales-palomo et al., 2017; Silva et al., 2018), and all four studies measuring ambulatory BP used an automated device (Ciolac et al., 2009; de Carvalho et al., 2014; Sosner et al., 2016; Ramirez-Jimenez et al., 2017). The remaining two studies used a manual device to measure office BP (Seeger et al., 2014; Pimenta et al., 2019) and one did not specify the device (de Carvalho et al., 2014). The intervention protocol of one study (Pimenta et al., 2019) was not sufficiently detailed to allow replication and was unclear in another study (Ramirez-Jimenez et al., 2017). All studies reported point and variability measures for BP and presented all BP data. As shown in Supplementary File 2 in Supplementary Material, visual inspection of the funnel plots did not reveal any publication bias. Duval and Tweedie's correction model were applied to the overall sample for both systolic and diastolic office BP; no trimmed studies were observed.

### Study and Participant Characteristics

Table 1 shows an overview of the study and participant characteristics. All studies were published between 2004 and 2019 and conducted in Brazil (*n* = 5) (Ciolac et al., 2009; de Carvalho et al., 2014; Costa et al., 2016; Silva et al., 2018; Pimenta et al., 2019), France (*n* = 3) (Mourot et al., 2004; Tordi et al., 2010; Sosner et al., 2016), Spain (*n* = 2) (Morales-palomo et al., 2017; Ramirez-Jimenez et al., 2017), the United States (*n* = 2) (Rossow et al., 2009; Angadi et al., 2015), the United Kingdom (*n* = 1) (Seeger et al., 2014), and New Zealand (*n* = 1) (Graham et al., 2016). Twelve studies used a randomized cross-over design (Mourot et al., 2004; Rossow et al., 2009; Tordi et al., 2010; de Carvalho et al., 2014; Seeger et al., 2014; Angadi et al., 2015; Costa et al., 2016; Graham et al., 2016; Morales-palomo et al., 2017; Ramirez-Jimenez et al., 2017; Silva et al., 2018; Pimenta et al., 2019) while the remaining two applied a randomized parallel design (Ciolac et al., 2009; Sosner et al., 2016)^.^ A total sample of 276 individuals (193 males; 83 females) was included in this meta-analysis. Five studies included only men (Mourot et al., 2004; Rossow et al., 2009; Tordi et al., 2010; Costa et al., 2016; Graham et al., 2016), and nine studies included both men and women (Ciolac et al., 2009; Rossow et al., 2009; Tordi et al., 2010; de Carvalho et al., 2014; Angadi et al., 2015; Costa et al., 2016; Sosner et al., 2016; Morales-palomo et al., 2017; Ramirez-Jimenez et al., 2017). Mean age of participants ranged from 22.5 to 69.5 years. Based on resting office BP, 10 study groups involved normotensive individuals (Mourot et al., 2004; Rossow et al., 2009; Tordi et al., 2010; Seeger et al., 2014; Angadi et al., 2015; Costa et al., 2016; Graham et al., 2016; Silva et al., 2018), four study groups included hypertensive patients (Rossow et al., 2009; Tordi et al., 2010; Costa et al., 2016; Pimenta et al., 2019), and four study groups included both normotensive and hypertensive participants (Morales-palomo et al., 2017; Ramirez-Jimenez et al., 2017).

**Table 1:** Overview of the general characteristics of the study and participants.

**Study(author, year, country)**	**Design**	**Subjects analyzed [(N) M + (N) F]**	**BP category**	**Age**	**Office SBP/DBP**	**Modality**	**HIIE characteristics**	**MICE characteristics**	**BP measurement (device)**	**Time point BP measurements**
**Ambulatory Blood Pressure**										
Carvalho et al.2014, Brazil (de Carvalho et al., 2014)	Crossover	8M/12F	Medicated Hypertensives	≥60 yrs	143.45 ± 10.18/88.50 ± 7.11 mmHg	Treadmill	**Time:** 42 min **Work-bout:** 4 min at the RCP **Recovery:** 2 min at 40%VO2peak	**Time:** 42 min **Intensity:** VAT	Ambulatory BP (Spacelabs® 90207)	20 h post-exercise
Ciolac et al.2009, Brazil (Ciolac et al., 2009)	Parallel	HIIE: 18M/8FMICE: 16M/10F	Medicated Hypertensives	HIIE:44 ± 9 yrsMICE:48 ± 7 yrs	HIIE: 129.3 ± 0.8/84.8 ± 6 mmHg MICE:129.9 ± 10/85.8 ± 10.4 mmHg	Cycle	**Time:** 30 min **Work-bout:** 2 min at 50% HRreserve **Recovery:** 1 min at 80%HRreserve	**Time:** 30 min **Intensity:** 60% HRres	Ambulatory BP (Spacelabs 90207)	24 h post-exercise
Sosner et al.2016, France (Sosner et al., 2016)	Parallel	HIIE: 9M/5FMICE: 8M/6F	High Normal/ Hypertensive	HIIE: 65 ± 8 yrsMICE:65 ± 6 yrs	HIIE:144.2 ± 17.3/87.6 ± 11.6 mmHg MICE:142.4 ± 11.4/81.9 ± 6.2 mmHg	Cycle	**Time:** 20 min **Work-bout:** 2 sets (10 min) of 15 s at 100% Power Output **Recovery:** 15 s of passive recovery (4 min passive recovery between the sets)	**Time:** 24 min **Intensity:** 50% Peak Power Output	Ambulatory BP (Mobil-O-Graph PWA)	24 h post-exercise
Ramirez-Jimenez et al.2017, Spain (Ramirez-Jimenez et al., 2017)	Crossover	G1:5M/3FG2:8M/3F	G1(*n* = 8): Normotensive G2 (*n* = 11): High normal/ hypertensive	G1: 53.3 ± 9.5yrsG2: 56.5 ± 6.2yrs	G1:116 ± 7/65 ± 7 mmHg G2:135 ± 17/86 ± 7mmHg	Cycle	**Total Time:** 28 min **Work-bout:** 4 × 4min at 90%HRpeak **Recovery:** 3 min at 70%HRpeak	**Time:** 53 ± 6 min **Intensity:** 60%HRpeak	Ambulatory BP (Oscar2, SunTech, Morrisville, NC, USA)	14 h post- exercise
**Office Blood Pressure**										
Angadi et al.2015, USA (Angadi et al., 2015)	Crossover	10M/1F	Normotensive	24.0 ± 3.7 yrs	122 ± 11/68 ± 7 mmHg	Cycle	**Total Time**: G1:28 min (HIIE near maximal) G2:15 min (HIIE supra-maximal) **Work bout:**4 × 4min at G1: 90–95%HRmax or G2: 6 × 30 s “all out” **Recovery:** G1: 3 min at 50%Hrmax or G2:4 min active recovery	**Time:** 30 min **Intensity:** 75–80% HRmax	Automatic Dinamap oscillometric BP monitor (GE Healthcare, Waukesha, WI, USA)	Every 15 min post-exercise for 3 h
Costa et al.2016, Brazil (Costa et al., 2016)	Crossover	14M	Normotensive	24.9 ± 4.1 yrs	120.5 ± 8.1/69.5 ± 6 mmHg	Treadmill	**Total Time:** 20 min **Work-bout:** 10 × 60 s at 90% MTV **Recovery:** 60 s at 30% of MTV	**Time:** 20 min **Intensity:** 60% MTV	Automatic - Oscillometric device (Omron®HEM-780-E, Kyoto,Japan)	Every 10 min for 60 min post- exercise
Graham et al.2016, New Zealand (Graham et al., 2016)	Crossover	12M	Normotensive	23 ± 3 yrs	116.3 ± 11.6/62.4 ± 9.4 mmHg	Cycle	**Total Time:** 20.3 min: 5 × 60 s (*all out)* **Recovery:** 4.5 min at 30W (legs) - 15W(arms)	**Time:** 50 min**Intensity:** 65% VO2max	Finometer (Finapress Medical Systems, The Netherlands)	30, 60, and 180 min post-exercise
Mourot et al.2004, France (Mourot et al., 2004)	Crossover	10M	Normotensive	24.6 ± 0.6 yrs	130.6 ± 7.1/71.7 ± 6.1 mmHg	Cycle	**Total Time:** 45 min **Work-bout:** 1 min peak work-rate **Recovery:** 4 min at base work-rate	**Time:** 45 min **Intensity:** power at 1st ventilatory threshold	Automatic- Office (BP-8800, Colin Electronics, Japan)	20 and 60 min post-exercise
Palomo et al.2017, Spain (Morales-palomo et al., 2017)	Crossover	11M/3F	G1 (*n* = 7): Hypertensive; G2(*n* = 7): Normotensive	Hypertensive: 59 ± 6 yrsNormotensive: 55 ± 9 yrs	Hypertensive: 135 ± 18.2/81 ± 7.9 Normotensive: 122.1 ± 9/75.2 ± 6	Cycle	**Time:** **~**460 kcal **Work-bout:** 5 × 4 min at 90%HRpeak **Recovery:** 3 min at 70%HRpeak	**Time:** ~460 kcal**Intensity:** 60% HRpeak for 70 ± 5 min	Automatic – Office (Tango™ SunTech Medical, Inc., Morrisville, NC, USA)	Pre-exercise and post-exercise
Pimenta et al.2019, Brazil (Pimenta et al., 2019)	Crossover	5M/15F	Medicated Hypertensive	51 ± 8 yrs	HIIE:127 ± 09/83 ± 08 mmHg MCE: 128 ± 15/83 ± 10 mmHg	Treadmill	**Time:** 30 min **Work-bout:** 5 × 3 min 85–95% of VO2reserve **Recovery:** 2 min active recovery at 50–60% VO2reserve	**Time:** 35 min **Intensity:** 60–70% VO2reserve	Manual	Every 10 min one single measure for 60 min post-exercise
Rossow et al.2010, USA (Rossow et al., 2009)	Crossover	15M/10F	Normotensive	25.5 ± 1.1	117 ± 8/63.4 ± 7 mmHg	Cycle	**Total Time:** 25 min **Work-bout:** 4 × 30 s “all-out” cycle sprint (~500W) **Recovery:** 4.5 min (<50rpm/30W)	**Time:** 60 min **Intensity:** 60% HRreserve	Automatic Oscillometric cuff (HEM-907 XL;Omrom, Shimane, Japan)	Post 30 min; Post 60 min
Seeger et al.2014, United Kingdom (Seeger et al., 2014)	Crossover	10M/7F	Normotensive	23 ± 4 yrs	121 ± 9/73 ± 8 mmHg	Cycle	**Total time:** 28 min **Work-bout:** 10 × 1 min at 100% Maximum workload **Recovery:** 2 min at 25% maximum workload	**Time:** 28 min **Intensity:** 50% of maximum workload	Manual	At 30 min post-exercise
Silva et al.2018, Brazil (Silva et al., 2018)	Crossover	23M	Normotensive	24.2 ± 2.8 yrs	118.2 ± 9.1/70.3 ± 7.0 mmHg	Treadmill	**Total Time:** 18 min **Work-bout:** 6 × 90 s at 80% VO2peak **Recovery:** 90 s at 40% Vo2peak	**Time:** 18 min **Intensity:** 40% VO2peak	Automatic –Sphygmomanometric device (OMROM – HEM 7200, Kyoto, Japan)	Every 10 min for 1 h post-exercise
Tordi et al.2010, France (Tordi et al., 2010)	Crossover	11M	Normotensive	22.5 ± 0.7 yrs	118.1 ± 4.8/65.5 ± 4.1 mmHg	Cycle	**Total Time:** 30 min **Work-bout:** 6 × 5 min 4 min at 65% HRmax **Recovery:** 1 min at 85% HRmax	**Time:** 30 min **Intensity:** the average HR achieved during HIIE	Automatic -Dinamap® GE Medical Systems, Bc, France	At 30 min post-exercise

*Data are reported as mean ± SD. BP, blood pressure; DBP, diastolic blood pressure; F, female; HIIE, high-intensity interval exercise; HR, heart rate; HRmax, maximal heart rate; M, male; MCE, moderate-intensity continuous exercise; Min, minutes; MVT, maximal treadmill velocity; N, number of participants; RCT, respiratory compensation threshold; s, seconds; SBP, systolic blood pressure; VAT, ventilatory anaerobic threshold; VO2, uptake oxygen*.

Ten studies (Mourot et al., 2004; Ciolac et al., 2009; Rossow et al., 2009; Tordi et al., 2010; Seeger et al., 2014; Angadi et al., 2015; Graham et al., 2016; Sosner et al., 2016; Morales-palomo et al., 2017; Ramirez-Jimenez et al., 2017) performed the exercise protocol on a cycle ergometer whereas the remaining four used a treadmill (de Carvalho et al., 2014; Costa et al., 2016; Silva et al., 2018; Pimenta et al., 2019). The exercise intensity was set as % of heart rate reserve (Ciolac et al., 2009), % of maximum heart rate (Tordi et al., 2010; Angadi et al., 2015; Morales-palomo et al., 2017; Ramirez-Jimenez et al., 2017), % of maximum load (Seeger et al., 2014; Costa et al., 2016), % of peak VO2 (de Carvalho et al., 2014; Silva et al., 2018), % of reserve VO2 (Pimenta et al., 2019), and power output (Sosner et al., 2016) or watts (Mourot et al., 2004; Rossow et al., 2009; Graham et al., 2016). MICE sessions lasted 18–65 min and HIIE lasted 15–45 min. Two studies reported that the HIIE and MICE bouts were isocaloric (~460 kcal) (Morales-palomo et al., 2017; Ramirez-Jimenez et al., 2017). The exercise intensity for MICE sessions ranged between 40 and 77.5% of heart rate (reserve and/or maximum). Within the HIIE sessions, the number of bouts ranged from 4 to 40, with 15 to 240 s of high-intensity activity interspaced with 15–270 s of active or passive recovery. The high-intensity bouts during HIIE ranged between 80 and 100%, while the intensity during active recovery was 70% of heart rate (reserve and/or maximum). Warm-up and cool-down time were excluded from the total duration of the sessions.

As shown in Table 1, office BP was measured pre-exercise and then 30 (*n* = 5 studies) (Rossow et al., 2009; Tordi et al., 2010; de Carvalho et al., 2014; Seeger et al., 2014; Morales-palomo et al., 2017) and 60 min (*n* = 8 studies) (Mourot et al., 2004; Angadi et al., 2015; Costa et al., 2016; Graham et al., 2016; Morales-palomo et al., 2017; Silva et al., 2018; Pimenta et al., 2019) post-exercise by means of an automated device (*n* = 6) (Rossow et al., 2009; Tordi et al., 2010; Costa et al., 2016; Graham et al., 2016; Ramirez-Jimenez et al., 2017; Silva et al., 2018), manual auscultatory method (*n* = 2) (Seeger et al., 2014; Pimenta et al., 2019) or by means of the Finapress (*n* = 1) (Graham et al., 2016). Four (Ciolac et al., 2009; de Carvalho et al., 2014; Sosner et al., 2016; Ramirez-Jimenez et al., 2017) studies used automatic ambulatory BP monitors of which three studies (Mourot et al., 2004; Ciolac et al., 2009; Sosner et al., 2016) reported 24 h ambulatory BP after exercise, four studies reported only day-time BP (Ciolac et al., 2009; de Carvalho et al., 2014; Sosner et al., 2016; Ramirez-Jimenez et al., 2017), and three studies (Ciolac et al., 2009; de Carvalho et al., 2014; Sosner et al., 2016) presented night-time BP.

### Office Blood Pressure

**Figure 3:**
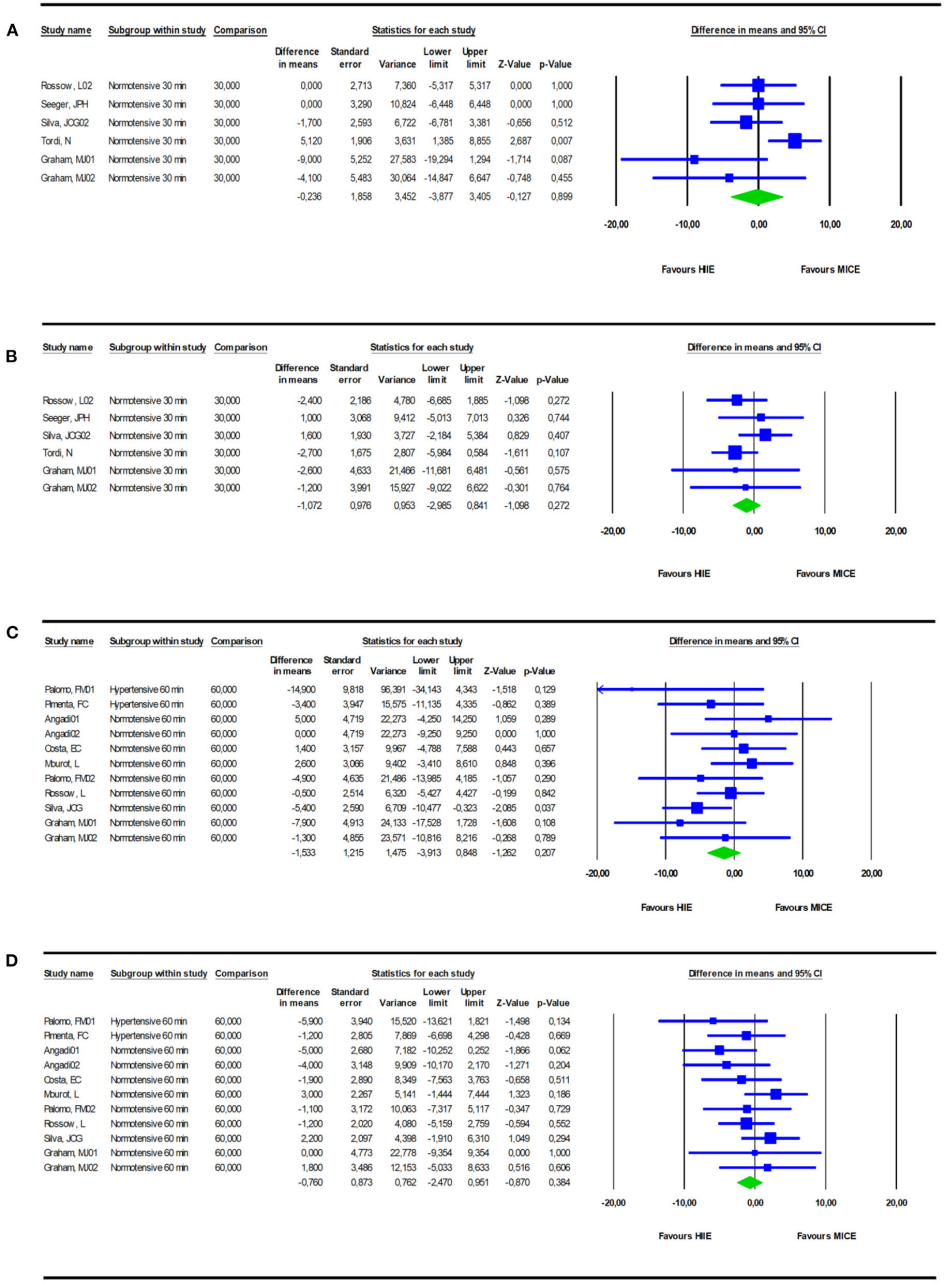
Comparison of PEH, measured as office BP at 30 [SBP **(A)**- DBP **(B)**] or 60 min [SBP **(C)**- DBP **(D)**] after completion of a bout of HIIE vs. a bout of MICE.

Pooling data from five studies (*n* = six study groups) evaluating systolic BP and diastolic BP 30 min following completion of the exercise sessions showed no difference between HIIE and MICE [−0.24 mmHg (−3.9 to +3.4; *I*^2^ = 52.3; *p* = 0.89)/−1.07 mmHg (−2.98 to +0.84); *I*^2^ = 0; *p* = 0.27] (Figures 3A,B). An *I*^2^ of 52.3% suggested high heterogeneity for systolic BP. Though, leave one out sensitivity analysis for systolic BP did not change the results (systolic BP ranged between +0.08 and −1.6; *p* > 0.30 for all). Similarly, pooled data from eight studies (11 study groups) found no differences in PEH between both exercise modalities 60 min after ending the session [−1.5 mmHg (−3.91 to +0.85); *I*^2^ = 12.77; *p* = 0.20/−0.76 (−2.47 to +0.95); *I*^2^ = 7.47%; *p* = 0.38] (Figures 3C,D). A sensitivity analysis omitting the two hypertensive subgroups did not change the results [−1.14 mmHg (–3.64 to +1.36); *I*^2^ = 13.1; *p* = 0.21/−0.43 mmHg (−2.3 to + 1.47); *I*^2^ = 10.3%; *p* = 0.22]. *I*^2^ < 15% suggested low heterogeneity. An overview of the BP changes at the individual study level is presented in Supplementary File 3 in Supplementary Material. Effect sizes calculated as SMD were small with pooled values of −0.037 and −0.12 for systolic and diastolic BP after 30 min recovery and −0.18 and −0.10 for systolic and diastolic BP after 60 min of recovery.

### Ambulatory Blood Pressure

**Figure 4:**
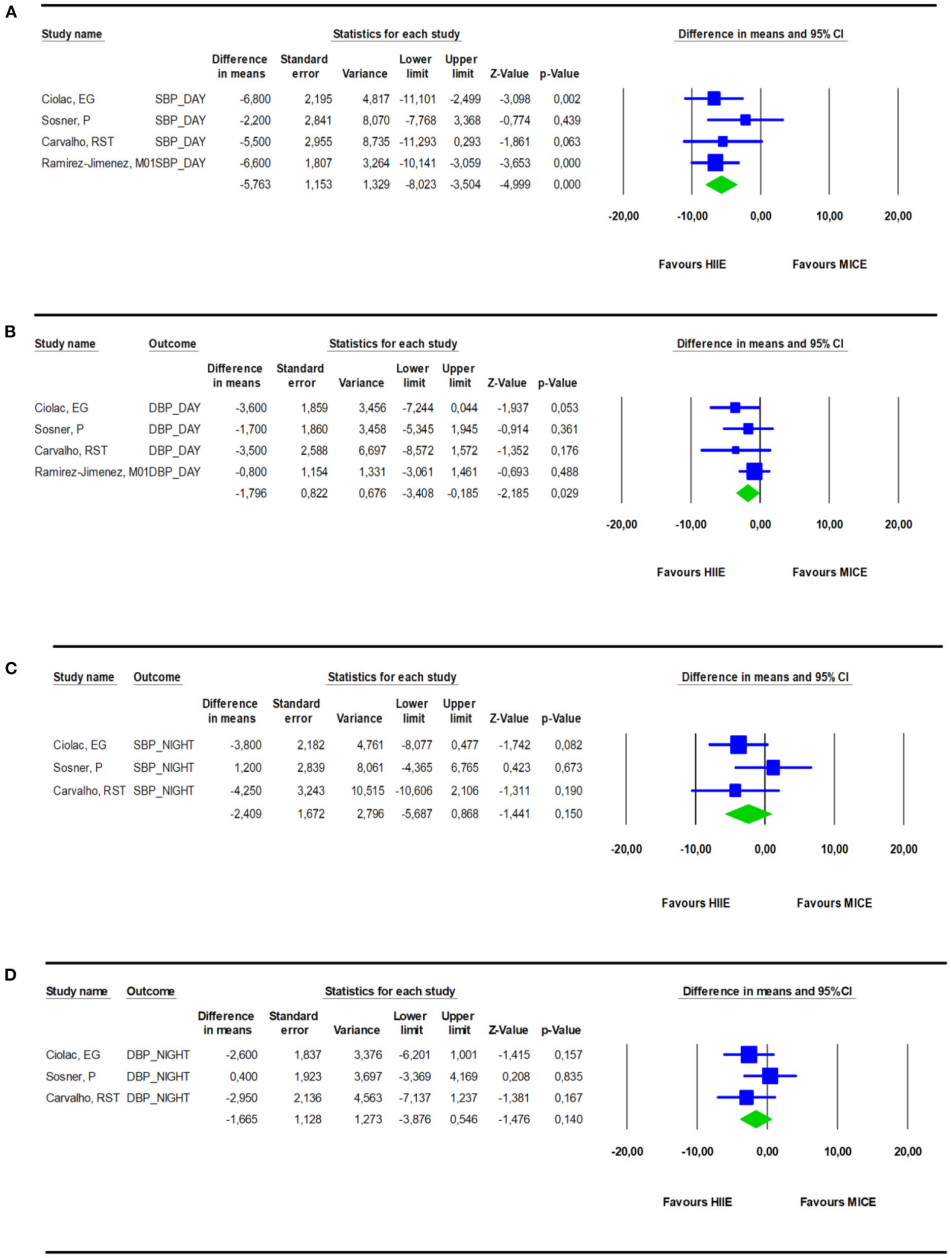
Effect of a bout of HIIE on daytime SBP **(A)** and DBP **(B)** monitoring compared to a bout of MICE. Effect of a bout of HIIE on nighttime SBP **(C)** and DBP **(D)** monitoring compared to a bout of MICE.

Compared with MICE, HIIE reduced daytime systolic and diastolic ambulatory BP by −5.3 mmHg (−7.3 to −3.3; *I*^2^ = 0%; *p* = < 0.001) (Figure 4A) and −1.63 mmHg (−3.00 to −0.26; *I*^2^ = 0%; *p* = 0.02), respectively (Figure 4B). However, nighttime ambulatory BP after HIIE was not significantly lower than MICE for both systolic [−2.4 mmHg (−5.7 to +0.87); *I*^2^ = 14.6; *p* = 0.1] (Figure 4C) and diastolic BP [−1.6 mmHg (−3.9 to +0.55); *I*^2^ = 0; *p* = 0.14] (Figure 4D). Heterogeneity was low for both daytime and nighttime BP. Three studies reported 24 h ambulatory BP and found no differences in systolic [−2.2mmHg (−5.9 to +1.48), *p* = 0.23] and diastolic BP [−0.76 (−4.0 to +2.51), *p* = 0.64] between HIIE and MICE, yet heterogeneity was high with *I*^2^ = 42% and *I*^2^ = 53%, respectively. An overview of the ambulatory BP changes at the individual study level is presented in Table 1. Pooled SMD showed a medium effect (−0.60) for daytime SBP whereas effect sizes for daytime DBP (−0.31) and nighttime SBP/DBP (−0.2/−0.185) were small.

## Discussion

The main aim of this systematic review and meta-analysis was to examine whether BP changes following a session of HIIE are more pronounced compared to a session of MICE. Findings of this meta-analysis suggest that a single session of HIIE is associated with a statistically significant and clinically meaningful larger reduction in daytime ambulatory BP compared to a single session of MICE.

Previous research suggested that exercise intensity had little impact on the manifestation of PEH when BP is measured for a short time period after exercise (Cornelissen and Fagard, 2004). Our results are in line with these older studies showing that office BP responses, measured in a quiet sitting or supine position, are not substantially different during the first 30 and 60 min after completion of a session of HIIE or MICE. Concordant to our results, a recent systematic review investigating acute cardiovascular response to HIIE also found that cardiovascular responses were quite similar within 1 h of completion of a session of HIIE or MICE (Price et al., 2020). In addition, meta-analytic research investigating the effect of a single session of dynamic resistance exercise on PEH (Casonatto et al., 2016) found no major impact of intensity on systolic and diastolic PEH after 60–90 min of exercise completion.

On the other hand, when PEH was analyzed by means of ambulatory BP monitoring during the daytime and/or nighttime hours following the exercise sessions, the present meta-analysis suggests that a single session of HIIE has a more pronounced and most likely longer lasting effect than a single session of MICE. Our findings are consistent with a previous study evaluating PEH over 9 h after single sessions of low, moderate, or vigorous exercise in 45 men (age 18–55 years) with elevated awake ambulatory BP, which found that although all exercise sessions (low, moderate, or vigorous) reduced the BP when compared to a non-exercise control session, the PEH occurred in a dose-response way with higher intensity exercise inducing a larger and more sustained BP reduction (Eicher et al., 2010). Previously, it was found that PEH following exercise has a strong correlation with BP lowering effect of chronic exercise training (Hecksteden et al., 2013). In that sense, our results are in contrast to the meta-analysis in adults of any health status performed by Way et al. (2019) who compared chronic effects (≥4 weeks) of both exercise interventions documenting a significant blood pressure reduction in favor of HIIE at night-time diastolic BP (−0.826 to −0.086 mmHg), and near significant difference for systolic BP day-time (−0.740 to 0.041 mmHg) and diastolic BP day-time (−0.717 to 0.020 mmHg).

Regarding the mechanisms associated with PEH, insufficient data did not permit us to quantitatively summarize potential differences in underlying mechanisms. In summary, Lacombe et al. (2011) showed that HIIE promoted greater changes in baroreflex sensitivity and HR variability compared to MICE. A single session of HIIE also promoted a larger reduction in stroke volume and a more pronounced increase in HR compared to MICE (Morales-palomo et al., 2017). Additionally, greater reductions on systematic vascular resistance and cutaneous vascular resistance have been observed following a single session of HIIE (Morales-palomo et al., 2017). In line, Costa et al. (2020) showed a significant decrease in systemic vascular resistance following HIIE compared to a control condition. The same authors (Costa et al., 2020) also found a lower vascular impedance after both MICE and HIIE sessions compared to the control session. The reduction in systematic vascular resistance, total vascular impedance, and pulse pressure, mainly after HIIE, might be explained by a sustained post-exercise vasodilation in the vascular beds of the lower limbs in treadmill exercise protocols (Costa et al., 2020). A complex interaction between neural and local vasodilatory mechanisms (e.g., sympathoinhibition due to baroreflex resetting, blunted transduction of sympathetic outflow to vasoconstriction, and histamine receptors activation) mediates the sustained post-exercise vasodilation (Halliwill et al., 2013; Hecksteden et al., 2013). During exercise, the likely higher increase of blood flow toward the active muscle following HIIE vs. MICE promotes increased shear stress (mechanical stimulus) on the endothelium, which mediates the release of vasodilatory substances, such as histamines, promoting a sustained vasodilatory response and reducing systemic vascular resistance (Halliwill et al., 2013; Hecksteden et al., 2013). Further studies should address these vasodilatory responses after different exercise intensities, as these mechanisms might explain in part a more sustained PEH following HIIE.

Further, the studies analyzing autonomic function reported higher heart rate post 30 min (Tordi et al., 2010), post the first hours (Morales-palomo et al., 2017), and post 20 h (de Carvalho et al., 2014) after HIIE than MICE sessions. This is in accordance with Abreu et al. (2019) in a systematic review (*n* = 193) who showed an improvement in parasympathetic and/or sympathetic modulation after HIIE (≥2 weeks) when evaluated by linear and non-linear indexes of heart rate variability (Abreu et al., 2019). Along with the improvement in endothelium responses as mentioned above (i.e., stimulating nitric oxide syntheses), the authors found HIIE superiority vs. MICE in cardiac autonomic variables due to greater degrees of distensibility of carotid artery which seems to be associated with improvements in baroreflex sensitivity, improving mitochondrial function and, consequently, capacity of skeletal muscle as well as improving maximal volume uptake, which may be correlated to the predominance of rest vagal modulation after HIIE (Abreu et al., 2019). On the other hand, Mourot et al. (2004) demonstrated that mean R-R interval measured by heart rate variability were lower 1 h after HIIE compared to MICE, but not post 24 or 48 h, suggesting that short-term heart rate variability depend on the type of exercise (i.e., intensity), contrary to the long-term recovery (i.e., total physical work performed during exercise) (Mourot et al., 2004).

### Limitations

This systematic review with meta-analysis has some limitations that need to be acknowledged. First, the number of randomized trials and their sample sizes was low. Moreover, studies evaluating office PEH shortly after exercise mainly involved normotensive individuals, whereas the four trials that assessed ambulatory BP included patients with hypertension under pharmacological treatment (two studies) or included only untreated individuals with stage 1 hypertension (two studies). Moreover, except for two trials, recruited participants were all younger than 60 years. In this context, one should be careful with generalizing present results to all hypertensive patients. We also observed a large variety of HIIE protocols, which ranged from the well-known Norwegian protocol of 4 × 4 min (Ramirez-Jimenez et al., 2017) to 10 × 1 min at 100% of maximal load (Seeger et al., 2014). Unfortunately, given the small number of studies, no subgroup analysis could be performed on the type of HIIE protocol. As only two trials reported that their exercise interventions were isocaloric, we cannot be 100% confident that the observed difference is due to a difference in intensity or a difference in volume. Therefore, this study emphasizes the need for more research investigating the role of HIIE on PEH and its mechanisms across all BP and age categories to maximize personalization of BP management for the growing group of older hypertensive patients.

## Conclusion

In summary, HIIE and MICE were similarly effective for promoting short-time PEH measured by office BP. On the other hand, HIIE showed larger PEH than MICE during daytime ambulatory BP monitoring. These findings suggest that HIIE may be a more time-efficient and beneficial antihypertensive tool compared to MICE. However, the number of studies assessing PEH by ambulatory BP was low and the office BP data were mainly derived from young normotensive/prehypertensive populations. Thus, future studies incorporating ambulatory BP monitoring, as well as including more hypertensive and older individuals, are needed to confirm HIIE's superiority as a safe BP lowering intervention in daily clinical practice.

## Data Availability Statement

The raw data supporting the conclusions of this article will be made available by the authors, without undue reservation.

## Author Contributions

VC, IM, KG, and RB contributed to conception and design of the manuscript. KG and IM performed data search and data extraction. JC, KG, and IM performed data-analysis. VC, IM, and KG drafted the manuscript. JC, EC, RB, VC, IM, and KG critically revised the manuscript. All authors contributed to the article and approved the submitted version.

## Conflict of Interest

The authors declare that the research was conducted in the absence of any commercial or financial relationships that could be construed as a potential conflict of interest.
