# Supplementary material for: Post-exercise Hypotension Following a Single Bout of High Intensity Interval Exercise vs. a Single Bout of Moderate Intensity Continuous Exercise in Adults With or Without Hypertension: A Systematic Review and Meta-Analysis of Randomized Clinical Trials
Source: Front Physiol. 2021 Jun 28;12:675289. doi: 10.3389/fphys.2021.675289 (PMC8274970; doi:10.3389/fphys.2021.675289)
Supplement: Supplementary file 1 [file Data_Sheet_1.docx]

***Supplementary File S1***

**Office systolic blood pressure (30 minutes)**

**Office diastolic blood pressure (30 minutes)**

**Office systolic blood pressure (60 minutes)**

**Office diastolic blood pressure (60 minutes)**

**Day-time ambulatory systolic blood pressure**

**Day-time ambulatory diastolic blood pressure**

**Night-time ambulatory systolic blood pressure**

**Nigh-time ambulatory diastolic blood pressure**
