# Supplementary material for: Post-exercise Hypotension Following a Single Bout of High Intensity Interval Exercise vs. a Single Bout of Moderate Intensity Continuous Exercise in Adults With or Without Hypertension: A Systematic Review and Meta-Analysis of Randomized Clinical Trials
Source: Front Physiol. 2021 Jun 28;12:675289. doi: 10.3389/fphys.2021.675289 (PMC8274970; doi:10.3389/fphys.2021.675289)
Supplement: Supplementary file 2 [file Table_1.docx]

***Supplementary File S2***

| Study | Clinical Status | Time BP (min) | N | Pre HIIE | Post HIIE | Pre MICE | Post MICE | Difference in means favor to HIIE (mmHg) | 95% CI |
| --- | --- | --- | --- | --- | --- | --- | --- | --- | --- |
| Graham MJ 01 et al, 2016^19^ | Normotensive | 30 | 12 | 119.1±10.5  62.4±11.8 | 104.8±11.1  57.2±10.4 | 117.4±12.0  64.0±8.6 | 112.1±16.3  61.4±13.1 | -9.0  -2.6 | -19.3 to 1.3  -11.7 to 6.5 |
| Graham MJ 02 et al, 2016^19^ | Normotensive | 30 | 12 | 112.6±12.4  60.8±8.4 | 103.2±11.8  57.0±6.5 | 117.4±12.0  64.0±8.6 | 112.1±16.3  61.4±13.1 | -4.1  -1.2 | -14.8 to 6.6  -9.0 to 6.6 |
| Rossow L02 et al, 2010^30^ | Normotensive | 30 | 25 | 117.8±8.5  63.9±7.5 | 117.6±12  59.3±9 | 116.4±7.5  62.9±6.5 | 116.2±9  60.7±7.5 | 0.0  -2.4 | -5.3 to 5.3  -6.7 to 1.8 |
| Seeger JPH et al 2015^34^ | Normotensive | 30 | 17 | 113±9.0  68±7.0 | 108±8.0  69±7.0 | 114±10.0  70±11.0 | 109±11.0  70±10.0 | 0.0  1.0 | -6.4 to 6.4  -5.0 to 7.0 |
| Silva JCG02 et al, 2018^31^ | Normotensive | 30 | 23 | 116.9±10.3  68.6±7.8 | 113.4±8.2  68.8±4.5 | 119.5±7.9  72±6.2 | 117.7±8.3  70.6±6.4 | -1.7  1.6 | -6.8 to 3.4  -2.4 to 5.4 |
| Tordi N et al, 2010^28^ | Normotensive | 30 | 11 | 113.9±4.7  66.4±4.5 | 112.1±4.4  63.2±3.7 | 122.3±4.9  64.7±4.2 | 115.4±3.5  64.2±2.7 | 5.12  -2.70 | 1.4 to 8.9  -6.0 to 0.6 |
| Random Model | Normotensive | 30 |  |  |  |  |  | -0.24  -1.1 | -3.9 to 3.4  -3.0 to 0.8 |
| Angadi SS01 et al, 2015^26^ (HIIT vs MCE) | Normotensive | 60 | 11 | 121±11.0  69±6.0 | 120±11.0  66±7.0 | 124±12.0  67±6.0 | 118±10.0  69±6.0 | 5.0  -5.0 | -4.2 to 14.2  -10.3 to 0.3 |
| Angadi SS02 et al, 2015^26^  (SIT vs MCE) | Normotensive | 60 | 11 | 124±11.0  68±9.0 | 118±11.0  66±8.0 | 124±12.0  67±6.0 | 118±10.0  69±6.0 | 0.0  -4.0 | -9.3 to 9.3  -10.2 to 2.2 |
| Costa EC et al, 201^29^ | Normotensive | 60 | 14 | 120.5±8.1  69.5±6 | 113±6  66.7±7.5 | 120.5±8.5  68.3±7.8 | 112.2±8.6  67.4±8.8 | 1.4  -1.9 | -4.8 to 7.6  -7.6 to 3.8 |
| Graham MJ 01 et al, 2016^19^ | Normotensive | 60 | 12 | 119.1±10.5  62.4±11.8 | 107.6±13.4  58.1±14.7 | 117.4±12.0  64.0±8.6 | 113.8±11.7  59.7±10.3 | -7.9  0.0 | -17.5 to 1.7  -9.4 to 9.4 |
| Graham MJ 02 et al, 2016^19^ | Normotensive | 60 | 12 | 112.6±12.4  60.8±8.4 | 107.7±11.4  58.3±5.4 | 117.4±12.0  64.0±8.6 | 113.8±11.7  59.7±10.3 | -1.3  1.8 | -10.8 to 8.2  -5.0 to 8.6 |
| Mourot L et al, 2004^15^ | Normotensive | 60 | 10 | 130,6±7.2  70.7±4.74 | 122.9±7.9  66.3±5.05 | 132.6±6.9  72.8±6.04 | 122.3±4.42  65.4±3.16 | 2.6  3.0 | -3.4 to 8.6  -1.4 to 7.4 |
| Palomo FM02 et al, 2017^16^ | Normotensive | 60 | 7 | 120.4±10.5  75.2±6.1 | 112.5±8.4  71.7±5.6 | 123.9±7.5  75.2±5.9 | 120.9±7.7  72.8±6.1 | -4.9  -1.1 | -14.0 to 4.2  -7.3 to 5.1 |
| Rossow L01 et al, 2010^30^ | Normotensive | 60 | 25 | 117.8±8.5  63.9±7.5 | 122.2±10.5  58.8±7.5 | 116.4±7.5  62.9±6.5 | 111.3±8.5  59±7 | -0.50  -1.20 | -5.4 to 4.4  -5.2 to 2.7 |
| Silva JCG01 et al, 2018^31^ | Normotensive | 60 | 23 | 116.9±10.3  68.6±7.8 | 111±7  70±7.4 | 119.5±7.9  72±6.2 | 119±8.9  71.2±6.9 | -5.40  2.20 | -10.5 to -0.3  -2.0 to 6.3 |
| Random Model | Normotensive | 60 |  |  |  |  |  | -1.1  -0.4 | -3.6 to 1.4  -2.3 to 1.5 |
| Random Model | Normotensive | 30 and 60 |  |  |  |  |  | -0.6  -0.7 | -2.7 to 1.5  -2.0 to 0.6 |
| Palomo FM01 et al, 2017^16^ | Hypertensive | 60 | 7 | 133.5±17.8  80.8±8.7 | 114±3.1  72.5±2.9 | 136.5±18.6  81.2±7.2 | 131.9±21.3  78.8±6.9 | -14.90  -5.90 | -34.1 to 4.3  -13.6 to 1.8 |
| Pimenta FC et al, 2019^17^ | Hypertensive | 60 | 20 | 127.1±9.2  83.5±7.6 | 117.9±12.1  82.6±8.4 | 127.8±14.7  82.7±10.7 | 122±12.8  83±8 | -3.40  -1.20 | -11.1 to 4.3  -6.7 to 4.2 |
| Random Model | Hypertensive | 60 |  |  |  |  |  | -5.6  -2.8 | -14.5 to 3.2  -7.3 to 1.7 |
| Random Model | Overall | Overall |  |  |  |  |  | -8.9  -0.9 | -3.0 to 1.2  -2.1 to 0.4 |

BP: blood pressure; Min: minutes; HIIE; high-intensity interval exercise; Hypertensive: individuals under anti-hypertensive treatment ; MICE: moderate-intensity continuous exercise; N: sample size; Normotensive: systolic and diastolic blood pressure ≥140/90mmHg, respectively.CI: confidence interval.
