# Supplementary material for: Post-exercise Hypotension Following a Single Bout of High Intensity Interval Exercise vs. a Single Bout of Moderate Intensity Continuous Exercise in Adults With or Without Hypertension: A Systematic Review and Meta-Analysis of Randomized Clinical Trials
Source: Front Physiol. 2021 Jun 28;12:675289. doi: 10.3389/fphys.2021.675289 (PMC8274970; doi:10.3389/fphys.2021.675289)
Supplement: Supplementary file 3 [file Table_2.docx]

***Supplementary file S3***

| Study | Group | N | Daytime  Post HIIE  SBP/DBP | Daytime  Post MICE  SBP/DBP | Difference in means favor to HIIE | CI95% | Night-time  Post HIIE | Night-time  Post MICE | Difference in means favor to HIIE | CI95% | 24h  Post HIIE | 24h  Post MICE | Difference in means | CI95% |
| --- | --- | --- | --- | --- | --- | --- | --- | --- | --- | --- | --- | --- | --- | --- |
| Carvalho, RST et al, 2014^18^ | Hypertensive | 20 | 122.5±6.8  73.5±5.3 | 128±6.2  77±5.6 | -5.50  -3.50 | -11.3 to 0.3  -8.6 to 1.6 | 110.15±8.9  62.1±4.5 | 114±8.7  65.05±5.9 | -4.25  -2.95 | -10.6 to 2.1  -7.1 to 1.2 |  | | | |
| Ciolac EG et al, 2009^32^ | Hypertensive | 52 | 127.1±2,1  83.3±7.1 | 128.3±9  84.2±10.1 | -6.80  -3.60 | -11.1 to -2.5  -7.2 to 0.0 | 113.5±9.4  70.1±5.9 | 111.7±8.7  70±10.2 | -3.80  -2.60 | -8.1 to 0.5  -6.2 to 1.0 | 123.2±8.7  79.5±6.1 | 122.7±5.6  79.7±10.4 | -5.10  -2.90 | -9.2 to -1.0  -6.5 to 0.7 |
| Mourot, L et al, 2004^15^ | Normotensive | 10 |  | | | | | | | | 127.7±5.0  68.8±2.8 | 128.4±3.4  67.9±5.8 | 1.30  3.0 | -4.1 to 6.7  -1.4 to 7.4 |
| Ramirez-Jimenez M01 et al, 2017^27^ | Hypertensive | 11 | 130.8±3.9  77.2±2.6 | 137.4±5.1  78±2.6 | -6.60  -0.80 | -10.1 to -3.0  -3.1 to 1.5 |  | | | | | | | |
| Ramirez-Jimenez M02 et al, 2017^27^ | Normotensive | 8 | 123.6±4.4  70.9±3 | 127.1±4.9  72.1±3.1 | -3.50  -1.20 | -7.9 to 0.9  -3.8 to 1.4 |  | | | | | | | |
| Sosner P et al, 2016^33^ | Hypertensive | 42 | 130.6±14.7  78.9±9.2 | 130.8±10.5  79.6±7.4 | -2.20  -1.70 | -7.8 to 3.4  -5.3 to 1.9 | 122.1±16.8  72.7±10.6 | 115.7±8.5  68.7±6.5 | 1.20  0.40 | -4.4 to 6.8  -3.4 to 4.2 | 127±14.1  75.9±9.4 | 125.2±8.8  75.3±6.5 | -1.90  -1.70 | -7.2 to 3.4  -5.3 to 1.9 |

CI: confidence interval; DBP: diastolic blood pressure; h: hour; HIIe: high-intensity interval exercise; Hypertensive: individuals in anti-hypertensive medication treatment; MICE: moderate-continuous intensity exercise; N: number of samples; Normotensive: without anti-hypertensive medication treatment; SBP: systolic blood pressure; SD: standard deviation.
